# Intraoperative monitoring of visual evoked potentials in patients undergoing transsphenoidal surgery for pituitary adenoma: a systematic review

**DOI:** 10.1186/s12883-021-02315-4

**Published:** 2021-07-23

**Authors:** Farizeh Jashek-Ahmed, Ivan Cabrilo, Jarnail Bal, Brett Sanders, Joan Grieve, Neil L. Dorward, Hani J. Marcus

**Affiliations:** Department of Neurosurgery, The National Hospital for Neurology and Neurosurgery, London, UK

**Keywords:** Transsphenoidal Surgery, Visual Evoked Potentials, Monitoring of Anterior Visual Pathway Function

## Abstract

**Background:**

Transsphenoidal surgery is the gold standard for pituitary adenoma resection. Although rare, a serious complication of surgery is worsened vision post-operatively.

**Objective:**

To determine whether, in patients undergoing transsphenoidal surgery for pituitary adenoma, intraoperative monitoring of visual evoked potentials (VEP) is a safe, reproducible, and effective technological adjunct in predicting postoperative visual function.

**Methods:**

The PubMed and OVID platforms were searched between January 1993 and December 2020 to identify publications that (1) featured patients undergoing transsphenoidal surgery for pituitary adenoma, (2) used intraoperative optic nerve monitoring with VEP and (3) reported on safety or effectiveness. Reference lists were cross-checked and expert opinion sought to identify further publications.

**Results:**

Eleven studies were included comprising ten case series and one prospective cohort study. All employed techniques to improve reliability. No safety issues were reported. The only comparative study included described a statistically significant improvement in post-operative visual field testing when VEP monitoring was used. The remaining case-series varied in conclusion. In nine studies, surgical manipulation was halted in the event of a VEP amplitude decrease suggesting a widespread consensus that this is a warning sign of injury to the anterior optic apparatus.

**Conclusions:**

Despite limited and low-quality published evidence regarding intra-operative VEP monitoring, our review suggests that it is a safe, reproducible, and increasingly effective technique of predicting postoperative visual deficits. Further studies specific to transsphenoidal surgery are required to determine its utility in protecting visual function in the resection of complex pituitary tumours.

## Introduction

Transsphenoidal surgery is the gold standard for pituitary adenoma resection, yet in one third of patients it is incomplete [1]. Advances in endoscopic surgery have opened possibilities for more complete tumour resections. However, the close relationship between the pituitary and the optic pathway implies that the benefits of complete resection must be balanced against the risk of post-operative visual dysfunction.

Visual-evoked potentials (VEP), as a means of intraoperative monitoring of visual function were first used during intra-orbital surgery in 1973 [2] and in our institution since 1985 [3] but have been criticised for being both unreliable and poorly reproducible and therefore not standardly adopted into common practice.

In recent years, the use of total intravenous anaesthesia (TIVA) [4, 5], the incorporation into the hardware of light-emitting diode (LED) technology [6–8] and adjuncts such as electroretinography (ERG) [6–8] and electroencephalography (EEG) [9] have attempted to overcome technical setbacks previously encountered in VEP neuromonitoring, enhancing the technique’s reproducibility and interpretability, and working to make it more reliable and easier to integrate into the operative workflow.

There are comparatively few reports of intraoperative VEP monitoring during transsphenoidal pituitary surgery despite the close relationship that this surgical approach maintains with the optic apparatus. Intuitively, the technique is best indicated for surgeries involving tumours of the anterior skull base that are particularly adherent to the optic chiasm and nerves such as craniopharyngiomas or meningiomas [10–13]. However, seeing the recent improvements in VEP monitoring technique facilitating its wider use, we sought to investigate the technique’s currently reported role during the far more common transsphenoidal procedure for pituitary adenoma.

Specifically, the aim of the present systematic review was to determine whether, in patients undergoing transsphenoidal surgery for pituitary adenoma, intraoperative visual evoked potential monitoring is a safe, reliable, and effective technological adjunct in intra-operatively alerting the surgeon of compromise to the anterior visual pathway, and in predicting post-operative visual outcome.

## Materials and Methods

### Search Methods

The PubMed and OVID platforms were searched over a 28-year period from January 1993 to December 2020 including the following databases: Books@Ovid, Journals@Ovid, CAB Abstracts, Embase, GeoRef, Medline, PsycINFO, Zoological Record Archive, and Zoological Record. The Boolean search term (pituitary OR hypophysectomy OR transsphenoidal OR endonasal OR Cushing’s OR ACTH OR acromegaly OR GH OR prolactinoma) AND ("visual evoked potential" OR VEP OR monitoring) AND (intraoperative) was used. Reference lists of included articles were also reviewed, and expert opinion sought, to identify further eligible publications. Two authors (FJA and JB) independently identified articles using the above search criteria. Expert opinion (IC) was sought to find additional papers.

### Inclusion and exclusion criteria

Titles and abstracts were screened to identify publications that (1) featured patients undergoing transsphenoidal surgery for pituitary adenoma, (2) used intraoperative VEP monitoring and (3) reported on safety or effectiveness. Full articles were obtained and further assessed for eligibility. Discrepancies were resolved by discussion with the senior author (HJM).

### Data extraction

The following data was extracted from eligible full articles: (1) study design, (2) study group characteristics including the number of patients and pathology, (3) VEP monitoring equipment details including mode of anaesthesia, (4) safety, (5) stability and reproducibility, and (6) effectiveness. With respect to the effectiveness of VEP, we considered the extent to which intra-operative VEP amplitudes and latencies allowed for prediction in post-operative visual waveform and visual function outcomes.

### Appraisal of evidence

The Methodological Index for Non-Randomised Studies (MINORS) scoring systems were used to guide evaluation of the quality of studies [14]. Studies of greater quality were given greater weighting in the qualitative analysis.

## Results

**Fig. 1:**
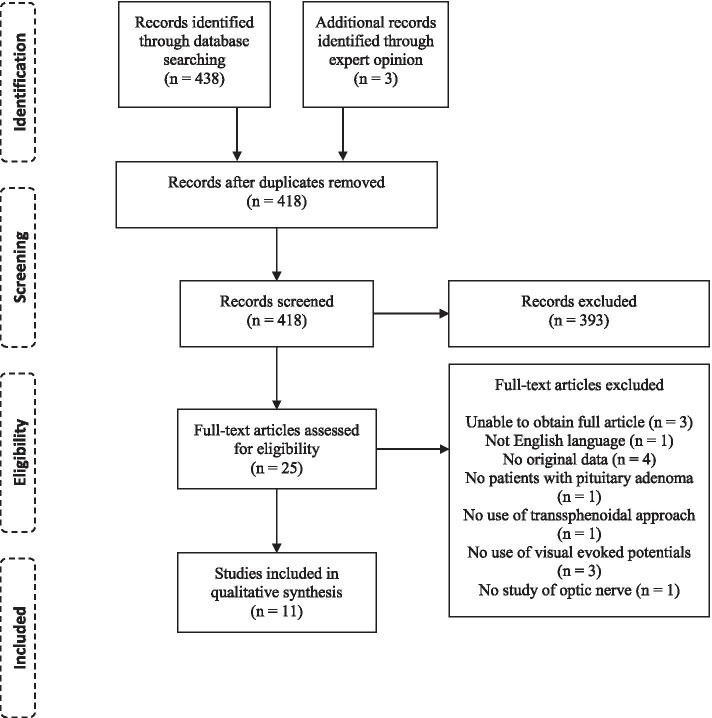
PRISMA flow diagram of article selection

A total of 438 articles were pooled from the electronic database (Fig. 1). Three further articles were identified via expert opinion. Of these, 23 articles were duplicates. 393 articles were excluded based on their title and abstract as they did not feature patients undergoing specifically transsphenoidal surgery for pituitary adenoma, did not include VEP monitoring, or did not report on safety or effectiveness.

Full text screening was conducted on the remaining 27 articles. This led to the exclusion of a further 14 articles. It was not possible to obtain the full text of three articles; one of these was an article from 1993 [15], two were conference abstracts only [16, 17]. A further case report was written in Japanese and not available in English [11]. Four articles did not present any original data. One was looking at cerebral aneurysms; one did not mention use of the transsphenoidal approach, three studies did not use visual evoked potentials and one was looking at the abducens nerve.

### Study design and study group characteristics

In all, 11 studies were identified that satisfied the inclusion criteria. These comprised one cohort study, and 10 case series (Table 1) [6, 7, 9, 18–25]. No randomised studies were found. It is important to note that only the cohort study by *Chacko *et al. looked exclusively at patients with pituitary adenoma. The remaining ten studies identified from the electronic database looked at patients undergoing all endoscopic surgery for sellar or parasellar tumours; whilst the bulk of this was pituitary adenoma, other pathology included meningioma, Rathke's cleft cyst, arachnoid cyst and craniopharyngioma amongst others. *Qiao *et al. observed and provided VEP analysis for 76 patients with sellar region tumours undergoing surgical decompression; their aim was to explore the use of artificial intelligence in VEP monitoring.

**Table 1:** Summary of included studies

Study	Study design	Patients	Number of patients with Pituitary Adenoma	Types of Pituitary Adenoma	Average size of tumour (cm)
Feng et al. (2019)^a^ [18]	Case series	42 pts with primary sellar neoplasms undergoing endoscopic TSS	40	Non-functioning: 32 GH-secreting: 5 Prolactinoma: 3	Non-secretory: 2.81 ± 1.10 Secretory: 3.29 ± 1.80 Other: 1.55 ± 0.64 Overall: 2.84 ± 1.23
Qiao et al. (2019) [19]	Case series	76 patients with sellar region tumour undergoing TSS (152 eyes tested)	Not reported	Not reported	Not reported
Toyama et al. (2018) [20]	Case series	20 pts undergoing endoscopic TSS (39 eyes tested)	16	Not reported	2.79 (range 1.5–4.5)
Nishimura et al. (2018) [21]	Case series	82 pts undergoing endoscopic TSS (164 eyes tested)	Not reported	Not reported	Not reported
Kurozumi et al.^a^ (2017) [22]	Case series	19 pts with sellar/parasellar tumours undergoing endoscopic TSS	17	Non-functioning: 14 GH-secreting: 3	Not reported
Luo et al. (2015) [23]	Case series	46 pts undergoing cranial surgery or TSS (85 eyes tested)	12	Not reported	Not reported
Kamio et al.^a^ (2014) [7]	Case series	33 pts with sellar or parasellar tumours undergoing TSS	25	Not reported	Not reported
Houlden et al.^a^ (2014 [9]	Case series	10 pts undergoing TSS for tumours near optic nerve or chiasm 2 pts undergoing craniotomy for an occipital lobe tumour and glial based tumour	Not reported	Not reported	Not reported
Chung et al. (2012) [24]	Case Series	53 pts with sellar or parasellar lesions undergoing endoscopic TSS (106 eyes tested)	37	Not reported	Not reported
Sasaki et al. (2010) [6]	Case Series	100 pts at intraoperative risk of visual impairment including 28 pts with parasellar lesions (200 eyes tested)	Not reported	Not reported	Not reported
Chacko et al. (1996) [25]	Cohort	36 pts undergoing TSS for pituitary adenomas; 22 with VEP monitoring, 14 without (72 eyes tested; 44 with VEP monitoring, 28 without)	36	Not reported	Not reported

*pts* patients, *TSS* Transsphenoidal surgery

^a^ These studies did not comment on individual eyes tested

The papers identified by expert opinion, *Luo *et al., *Houlden *et al. and *Sasaki et al.,* describe a broader use of VEPs in neurosurgery and therefore also include patients undergoing craniotomy; where possible, information specifically related to patients undergoing transsphenoidal surgery for pituitary adenoma has been extracted from these.

The quality of the included studies was variable (Table 2). The only comparative study reviewed was also the oldest. Performed by *Chacko *et al. in 2009 it is of fair quality (MINORS 16/24). Limitations are a lack of mention of consecutive patients, blinding, prospective calculation of study size, and length of follow up. The remaining studies were of similar quality (MINORS SCORE ranging 9/16 to 13/16). The highest quality of these were the studies by *Chung *et al.,* Feng *et al. and *Toyama *et al. (MINORS 13/16, 12/16 and 12/16 respectively). None of these studies documented a prospective calculation of study size. There was variability in the inclusion of consecutive patients, unbiased assessment of the study end-point and adequate follow-up period.

**Table 2:** Quality of studies using MINORS criteria

Study (year)	Clearly stated aim	Inclusion of consecutive patients	Prospective collection of data	Endpoints appropriate to the aim of the study	Unbiased assessment of the study endpoint	Follow-up period appropriate to the aim of the study	Loss to follow up less than 5%	Prospective calculation of the study size	An adequate control group	Contemporary groups	Baseline equivalence of groups	Adequate statistical analysis	TOTAL
Feng et al. (2019) [18]	2	2	2	2	1	1	2	0	n/a	n/a	n/a	n/a	12/16
Qiao et al. (2019) [19]	2	0	1	2	1	2	2	0	n/a	n/a	n/a	n/a	10/16
Toyama et al. (2018) [20]	2	2	2	2	1	1	2	0	n/a	n/a	n/a	n/a	12/16
Nishimura et al. (2018) [21]	2	1	2	1	1	1	2	0	n/a	n/a	n/a	n/a	10/16
Kurozumi et al. (20187) [22]	1	2	1	2	1	1	2	0	n/a	n/a	n/a	n/a	10/16
Luo et al. (2015) [23]	2	2	1	1	1	2	1	0	n/a	n/a	n/a	n/a	10/16
Kamio et al. (2014) [7]	2	2	2	1	0	2	2	0	n/a	n/a	n/a	n/a	11/16
Houlden et al. (2014) [9]	1	0	2	1	2	1	2	0	n/a	n/a	n/a	n/a	9/16
Chung et al. (2012) [24]	2	2	2	2	2	2	1	0	n/a	n/a	n/a	n/a	13/16
Sasaki et al. (2010) [6]	2	0	2	1	0	2	2	0	n/a	n/a	n/a	n/a	9/16
Chacko et al. (1996) [25]	2	0	2	2	0	0	2	0	2	2	2	2	16/24

### VEP monitoring equipment

The manufacturer and monitoring device used to analyse VEP waveform was mentioned in all studies except by *Feng *et al*.* The remaining ten studies used a combination of eight different signal processors (Table 3).

**Table 3:** Visual evoked potential signal processing devices used in each study

Study (Year)	VEP SIGNAL PROCESSING DEVICE	
Feng et al. (2019) [18]	Not reported	
Qiao et al. (2019) [19]	NIM-ECLIPSE	(MEDTRONIC, USA)
Toyama et al. (2018) [20]	Neuropack X1 MEB 2312 MEE 1232,	(NIHON KOHDEN, Japan) (NIHON KOHDEN, Japan)
Nishimura et al. (2018) [21]	MEE 1232,	(NIHON KOHDEN, Japan)
Kurozumi et al. (2017) [22]	NIM-ECLIPSE E4	(MEDTRONIC, USA)
Luo et al. (2015) [23]	ISIS System	(INOMED, Germany)
Kamio et al. (2014) [7]	Neuropack X1 MEB-2312,	(NIHON KOHDEN, Japan)
Houlden et al. (2014) [9]	Cadwell Elite intraoperative monitoring machine	(CADWELL INSTRUMENTS, USA)
Chung et al. (2012) [24]	PROTEKOR TM 10 M	(XLTEK, Canada)
Sasaki et al. (2010) [6]	Synax 1100 Neuropack	(NEC MEDICAL SYSTEMS, USA) (NIHON KOHDEN, Japan)
Chacko et al. (1996) [25]	Brain Atlas III system	(BIOLOGIC SYSTEMS, USA)

#### Mode of anaesthesia

*Kurozumi *et al. did not comment on anaesthetic regimen used*.* The majority of the remaining studies reported the use of total intra-venous anaesthesia (TIVA) (Table 4). 3 studies utilised Bispectral Index (BIS) monitoring to maintain the depth of anaesthesia between BIS-values of 40–60 [7, 20, 24]. These values represent the recommendation given by the National Institute for Health and Care Excellence (NICE) [26]. *Feng *et al. describe one exclusion for an unreliable VEP secondary to anaesthetic regimen. *Houlden *et al. commented on the use of simultaneous EEG.

**Table 4:** Methods used to promote stability of visual evoked potential waveform

Study (Year)	VEP waveform reproducibility / stability	Mode of Anaesthesia	Stimulus delivery device	Simultaneous ERG Monitoring
Feng et al. (2019) [18]	Not reported	TIVA	Flexible silicone patch LED goggles	No
Qiao et al. (2019) [19]	Not reported	TIVA	Light-proof goggles with a flashing LED	No
Toyama et al. (2018) [20]	97%	TIVA	Round silicone disc embedded with 16 red high luminosity flashing (100mCd) LEDs	Yes
Nishimura et al. (2018) [21]	98%	TIVA	Silicon discs with 16 red LEDs (100mCd)	Yes
Kurozumi et al. (2017) [22]	100%	Not reported	2 cm round silicone disc embedded with 16 red high luminosity flashing (100mCd) LEDs	Yes
Luo et al. (2015) [23]	83%	TIVA	Transparent eye patches placed on the closed eyes. Then the light-stimulating device was placed on the eyelids and covered with another transparent eye patch	No
Kamio et al. (2014) [7]	85%	TIVA	2 cm soft silicone disc embedded with 16 red high luminosity flashing (100mCd) LEDs	Yes
Houlden et al. (2014) [9]	83%	TIVA/ Gas inhalation	Goggle 3000mCd LED stimulators (3 LEDs on each side)	No
Chung et al. (2012) [24]	90%	TIVA	Bright LED goggles (XLTEK, Ontario, Canada)	No
Sasaki et al. (2010) [6]	94%	TIVA	2 cm silicone disc embedded with 16 red high-luminosity (100mCd) LEDs	Yes
Chacko et al. (1996) [25]	Not reported	Gas inhalation	Red LEDs fitted on goggles	No

*LED* Light-emitting diode

*Chacko *et al. was the only study to use solely gas anaesthesia; a combination of 60% nitrous oxide and 0.5% halothane with muscle relaxants and morphine. As gas anaesthesia is thought to cause VEP instability, they attempted to reduce this by taking baseline recordings more than 30 min after the induction of anaesthesia. *Houlden *et al. also used inhalation agents for two of their patients and whilst they found they could initially maintain a stable VEP, the reproducibility was subsequently impaired by bolus injections of propofol and a high MAC of desflurane.

#### Light stimulus delivery device

Five of the studies report the use of LED goggles for stimulus delivery (Table 4). The study by *Chung *et al. names the manufacturer as XLTEK (Ontario, Canada.) *Feng *et al. report two exclusions due to technical malfunctions of the optic goggles.

*Luo *et al. describe the placement of a light stimulating device between two transparent eye patches on top of the eye.

In their study, *Sasaki *et al. introduced a 2 cm round silicone disk embedded with 16 red high luminosity (100mCd) LEDs to reduce light axis deviation from frontal scalp-flap reflection. The remaining four studies all describe the use of this method [7, 20–22]. *Toyama *et al. also used a black light shield patch on the device to avoid interference between light stimulations. No other study commented on this.

#### ERG monitoring

ERG confirms the arrival of adequate light stimulation at the retina. The use of simultaneous ERG monitoring was reported by five of the studies (Table 4). *Toyama *et al. report one case of intra-operative wire breakage leading to loss of ERG signal.

### Safety

There were no cases of operative mortality reported in the any of the studies or any operative complications directly related to intra-operative VEP monitoring. *Kamio *et al. reported three cases of detachment of the VEP recording electrode from its occipital position but this did not result in any adverse effects. The device introduced by Sasaki, and utilised by several others, *“incorporates a safety system that shuts it down if continuous illumination by the LEDs exceeds [four]seconds” *[6]*.*

There was no report of any pressure-related eye problems from the goggles or silicone disk in any study.

### Stability and reproducibility

All studies commented on the importance of obtaining stable and reproducible VEPs and corresponding data was elicited from nine of the studies (Table 4). This ranged from 83–100%.

Stability was assessed in several studies by performing serial VEP recordings at baseline, after induction of anaesthesia, and then continuously intra-operatively. *Qiao *et al. commented that there was a significant decrease in amplitude after anaesthesia and a non-significant increase in latency.

Most studies confirmed reproducibility of the VEP waveform by at least two consecutive recordings prior to commencement of surgical manipulation [7, 9, 21–24].

There was consensus that VEP recordings can only be obtained in patients without severe visual impairments and the best corrected visual acuity in patients in whom reproducible VEP responses were recorded ranged from 0.2 – 0.4 [21, 24]. *Toyama *et al. were unable to record pre-operative VEP in one patient with severe pre-operative visual impairment and *Nishimura *et al., *Kamio *et al. and *Houlden *et al. excluded two patients in each of their studies for a similar reason. *Luo *et al. obtained VEP in all eyes with intact preoperative visual function but only 56% of cases with impaired vision. Reproducible VEP responses could not be obtained in 11/106 eyes and 12 eyes out of 100 patients in the studies by *Chung *et al. and *Sasaki *et al. respectively.

VEP stability and reproducibility are also affected by non-patient factors and particularly important techniques to augment this are the use of total intra-venous anaesthetic (TIVA), the use of LED goggles or silicone discs for light stimulus delivery, a black shield patch placed over the eyes and braided electrode cables, [20] and the use of simultaneous electroretinography (ERG) monitoring [12]. One or more of these techniques were employed in all studies (Table 4). Accordingly technical malfunctions secondary to anaesthetic regimen, [9, 18] malfunction of optic goggles [18] and detachment of the VEP recording electrodes [7] were documented causes of unobtainable or unreliable VEP. Low amplitude EEG may also have a role in maintaining VEP stability as suggested by *Houlden *et al. However, this technique was not used in any of the other studies.

#### VEP amplitudes

VEP amplitudes were monitored throughout the operation in all studies. The changes observed in baseline VEP amplitude were described in all the case series except *Houlden *et al. (Table 5). Apart from *Chung *et al., the remaining studies commented on whether the baseline VEP amplitude remained unchanged or whether it showed an improvement, temporary deterioration or permanent deterioration. *Chung *et al. did not comment on whether VEP amplitude deterioration was temporary or permanent.

**Table 5:** Intra-operative changes in Visual-Evoked Potential Waveforms

Title	Unchanged	VEP Improvement	Temporary VEP deterioration	Permanent VEP deterioration
Feng et al. (2019) [18]	73.8%	2.4%	14.3%	9.5%
Qiao et al. (2019) [19]^a^	24.3%	44%	30%	0
Toyama et al. (2018) [20]	53.8%	7.6%	23.1%	15.4%
Nishimura et al. (2018) [21]	77.5%	n/a	16.3%	5.0%
Kurozumi et al. (2017) [22]	89.5%	5.9%	5.9%	0
Luo et al. (2015) [23]	72%	16%	20%	12%
Kamio et al. (2014) [7]	82.1%	0%	14.3%	3.6%
Houlden et al.(2014) [9]	-	-	-	0
Chung et al. (2012) [24]	67.4%	20.0%	-	12.6%^c^
Sasaki et al. (2010) [6]	90.3%	0.5%	1.6%	7.5%
Chacko et al. (1996)^b^ [25]	-	-	100%	-

*VEP* Visual-Evoked Potential

Standard criteria for changes in VEP amplitude: Improvement > 50% increase in baseline VEP amplitude; Deterioration > 50% decrease in baseline VEP amplitude. Exceptions: ^a^:*Qiao* et al > 25% increase in baseline VEP amplitude; Deterioration > 25% decrease; ^b^Chacko et al – criteria not defined

^c^Did not report whether this deterioration was temporary or permanent

Most studies reported the same criteria for measuring changes in VEP amplitude: For improvement—a greater than 50% increase in baseline VEP amplitude; and for deterioration—a greater than 50% decrease in baseline VEP amplitude was required [18, 20–22, 24]. The exceptions were the studies by *Qiao* et al., who used 25% instead and *Chacko *et al. who did not define criteria.

All studies reported that in the event of a VEP amplitude deterioration, the surgeon was alerted, and surgical manipulations were stopped temporarily.

The VEP waveforms remained unchanged in the majority of operations across all the studies (24–90%). VEP deterioration was more often reported to be temporary than permanent. Permanent VEP deterioration ranged from 0 – 15%. Six studies showed an improvement in VEP waveform [18, 20, 24]. Of note, the study by *Qiao *et al. demonstrated a lower rate of unchanged VEP and a higher rate of both VEP improvement and temporary deterioration than the other studies which may be attributed to the lower thresholds they used.

In the cohort study by *Chacko *et al., all eyes in the testing group (with VEP monitoring) exhibited a transient decrease in VEP amplitude. This was also noted in the study by *Houlden *et al. in relation to amplifier blocking caused by electrocautery.

#### VEP latency

VEP latency was specifically examined alongside amplitude as a further parameter for evaluating VEPs in six of the studies. Several changes were observed although none of these were deemed to be statistically significant. *Feng *et al. described no significant association between latency and visual field outcomes. *Qiao *et al. found VEP latency increased after anaesthesia and Luo et al.found no statistically significant differences in latency between groups with different baseline pre-operative visual function. *Kamio *et al. and *Chung *et al. reported the mean VEP latencies but did not correlate these to visual function. In the study by *Sasaki *et al., only one case of prolonged latency was described out of 14 eyes which demonstrated permanent deterioration and no changes of latency were described in the 3 eyes which demonstrated temporary deterioration. Both *Chung *et al. and *Sasaki *et al. commented that latency is difficult to evaluate in the presence of decreased amplitude.

### Effectiveness

Here we define effectiveness as the capacity of intra-operative VEPs to predict visual function outcomes. All the studies except *Houlden *et al. and *Qiao *et al. commented on this.

Most of the studies looked at the visual outcomes pre- and post-operatively by looking at both visual acuity and visual fields. *Nishimura *et al.,* Luo *et al., and *Sasaki *et al. did not report on these separately but commented on outcomes of post-operative visual function which took both into consideration. *Table *6 provides an overview of associations observed between changes in VEP and patient visual outcomes. *Qiao *et al. did not measure post-operative visual outcomes as this was not the focus of their study and acknowledge this as a limitation to their work.

**Table 6:** Relationship between intra-operative visual-evoked potential (VEP) and post-operative visual function

**Study (Year)**	**Intra-operative VEP**	**Post-operative** **Visual Acuity n (%)**			**Post-operative** **Visual Fields n (%)**			**Notes**
		**Improved**	**Stable**	**Worsened**	**Improved**	**Stable**	**Worsened**	
**Feng et al. (2019) ** [18]	Improved	0 (0)	1 (100)	0 (0)	0 (0)	1 (100)	0 (0)	Sensitivity and specificity of VEP amplitude in detecting changes in visual field outcomes are 75% and 79%, respectively
	Stable	27 (87)		4 (13)	30 (97)		1 (3)	
	Worsened (transient)	1 (17)	2 (33)	3 (50)	2 (33)	3 (50)	1 (17)	
	Worsened (permanent)	2 (50)	1 (25)	1 (25)	1 (25)	1 (25)	2 (50)	
**Qiao et al. (2019)** [19]								Did not measure post-operative outcomes
**Toyama et al. (2018) ** [20]	Improved	3 (100)	0 (0)	0 (0)	1 (33)	2 (66)	0 (0)	No significant relationship observed between VEP and visual field outcome
	Stable	11 (53)	10 (48)	0 (0)	9 (43)	12 (57)	0 (0)	
	Worsened (transient)	6 (66)	3 (33)	0 (0)	5 (56)	4 (44)	0 (0)	
	Worsened (permanent)	3 (50)	3 (50)	0 (0)	1 (17)	5 (83)	0 (0)	
**Kamio et al. (2014)** [7]	Improved	0 (0)	0 (0)	0 (0)	0 (0)	0 (0)	0 (0)	No statistical analysis performed
	Stable	11 (48)	12 (52)	0 (0)	5 (22)	18 (78)	0 (0)	
	Worsened (transient)	2 (50)	2 (50)	0 (0)	2 (50)	2 (50)	0 (0)	
	Worsened (permanent)	0 (0)	1 (100)	0 (0)	0 (0)	0 (0)	1 (100)	
**Houlden et al. (2014)** [9]								Did not measure post-operative outcomes
**Chung et al (2012)** [24]	Improved	4 (22)	13 (68)	2 (11)	13 (68)	4 (22)	2 (11)	No association found between intraoperative VEP waveforms and post-operative visual acuity or visual fields
	Stable	13 (20)	42 (66)	9 (14)	39 (64)	17 (26)	8 (13)	
	Worsened (transient /permanent)	5 (42)	5 (42)	2 (17)	6 (50)	3 (25)	3 (25)	
**Chacko et al. (1996)** [25] **Group A**	Improved	0 (0)	0 (0)	0 (0)	0 (0)	0 (0)	0 (0)	There was statistically significant improvement in post-operative visual field testing between the test group A (with VEP monitoring) and the control group B (without VEP monitoring) There was no statistical difference in visual acuity between the two groups
	Stable	0 (0)	0 (0)	0 (0)	0 (0)	0 (0)	0 (0)	
	Worsened (transient)	n/a	n/a	n/a	34 (77)	10 (23)	0 (0)	
	Worsened (permanent)	0 (0)	0 (0)	0 (0)	0 (0)	0 (0)	0 (0)	
		**Post-operative Visual Function**^a^						
		**Improved n (%)**		**Stable n (%)**		**Worsened n (%)**		
**Nishimura et al. (2018)** [21]	Improved	0 (0)		0 (0)		0 (0)		“Intraoperative monitoring of VEP predicts postoperative visual function, and a reversible change in VEP indicates that visual function will be preserved”(2)
	Stable	62 (50)		62 (50)		0 (0)		
	Worsened (transient)	8 (31)		18 (69)		0 (0)		
	Worsened (permanent)	0 (0)		8 (100)		0 (0)		
**Luo et al.**^b^**(2015)** [23]	Improved	0 (0)		0 (0)		0 (0)		Intra-operative VEP has a specificity of 96% and a negative predictive value of 90% in detecting post-operative visual functionPreservation of VEPs predicted preserved visual function
	Stable	8 (16)		36 (72)		6 (12)		
	Worsened (transient)	0 (0)		10 (100)		0 (0)		
	Worsened (permanent)	0 (0)		2 (2)		0 (0)		
**Sasaki et al.**^b^** (2010) ** [6]	Improved	1 (100)		0		0 (0)		“Changes in intraoperative VEP findings, especially in the VEP amplitude, were well correlated with postoperative visual function”(3)
	Stable	17 (10)		150 (89)		2 (1)		
	Worsened (transient)	1 (33)		2 (67)		0		
	Worsened (permanent)	0		0		14 (100)		
**Kurozumi et al. (2017)** [22]								80% of patients with pre-operative visual disturbances had an improved visual acuity immediately after surgery 80% of patients had improved visual fields immediately after surgery

^a^Looked at visual function (if either or both of visual acuity/visual fields were improved, unchanged or worse then the outcome was considered improved, unchanged or worse)

^b^It was not possible to separate the data of the patients undergoing transsphenoidal surgery from other approaches in these studies

The studies by *Feng *et al., *Sasaki *et al.,* Kamio *et al. and *Luo *et al. suggested that there was a correlation between intra-operative VEP changes and post-operative visual outcomes. The study by *Feng *et al. describes this association with visual fields whereas *Luo *et al. and *Sasaki *et al. describe an association with visual function as a whole and do not distinguish between visual fields and acuity. *Toyama *et al., *Chung *et al. and *Chacko *et al. found no association. *Nishimura *et al. speculate in favour of a correlation between the two.

Table 7 calculates the sensitivity, specificity, positive predictive value and negative predictive value of each these studies from the data provided. Where visual field and visual acuity data was reported separately [7, 18, 20, 24, 25] the visual field outcomes only were used to calculate new post-operative visual deficit as visual fields have been found to be the more consistent measure of post-operative visual function for VEP monitoring in the literature. In the studies by Nishimura et al., Luo et al., Sasaki et al. and *Kurozumi *et al., whilst post-operative visual outcome data was reported, this was not split into visual field and visual acuity therefore the figures provided have been used.

**Table 7:** Sensitivity, specificity, positive predictive value and negative predictive value of VEP amplitude in predicting visual function outcomes^a^

Study	True Positive^b^ (TP)	False Positive^c^ (FP)	True Negative^d^ (TN)	False Negative^e^ (FN)	Sensitivity^f^ (Sn)	Specificity^g^ (Sp)	Positive Predictive Value^h^ (PPV)	Negative Predictive Value^i^ (NPV)
Feng et al. (2019) [18]	1	1	37	3	25%	97%	50%	93%
Qaio et al. (2019) [19]	-	-	-	-	-	-	-	-
Toyama et al. (2018) [20]	0	6	33	0	n/a	85%	n/a	100%
Nishimura et al. (2018) [21]	0	8	150	0	n/a	95%	n/a	100%
Kurozumi et al. (2017) [22]	-	-	-	-	-	-	-	-
Luo et al.^k^ (2015)[23]	0	2	54	6	n/a	96%	n/a	90%
Kamio et al. (2014) [7]	1	0	27	0	100%	100%	100%	100%
Houlden et al. (2014) [9]	-	-	-	-	-	-	-	-
Chung et al.^j^ (2012) [24]	-	-	-	-	-	-	-	-
Sasaki et al.^k^ (2010) [6]	14	8	171	2	88%	96%	64%	99%
Chacko et al. (1996) [25]	0	0	44	0	n/a	100%	n/a	100%

^a^ Where possible, visual field outcomes only were used to predict visual function outcomes. In the studies by Nishimura et al., Luo et al., Sasaki et al. and Kurozumi et al. this was not possible therefore combined visual acuity/field data has been used. ^b^TP = a permanent decrease in VEP amplitude and new post-operative visual deficit. ^c^FP = a permanent decrease in VEP amplitude but no new post-operative visual deficit. ^d^TN = no permanent decrease in VEP amplitude and no new post-operative visual deficit. ^e^FN = no permanent decrease in VEP amplitude but new post-operative visual deficit. ^f^Sn = TP/ (TP + FN) ^g^Sp = TN (TN + FP) ^h^PPV = TP / (TP + FP)^i^ NPV = TN/ (TN + FN) ^j^Unable to derive figures for Chung et al. as they combined transient and permanent VEP amplitude loss in their results. ^k^It was not possible to separate the data of the patients undergoing transsphenoidal surgery from other approaches in these studies; figures demonstrated are for all approaches

*Feng *et al. found a direct correlation between intra-operative VEP changes – specifically amplitude – and post-operative changes in visual fields, with an odds ratio of 3.15 (95% CI 1.15–8.59). They calculated the sensitivity and specificity of VEP amplitude in detecting changes in visual field outcome as 75% and 79% respectively.

*Luo *et al. calculated the association between intra-operative VEP and post-operative visual function to have a specificity of 96% [88–100%] and a negative predictive value (NPV) of 90% [79–96%] but reported the positive predictive value (PPV) could not be calculated because there was no true positive (TP) loss of VEP in their series. These statistics were influenced by the three patients who developed homonymous hemianopia post-operatively without any change in intra-operative VEP. It must be noted that these three patients did not have pituitary tumours and these changes reflect a failure to detect changes in the posterior visual pathway. They did comment that intraoperative VEPs were sensitive enough to detect mechanical manipulation of the anterior visual pathway in an early reversible stage.

*Toyama *et al. studied 39 eyes of which none experienced a worsening in visual acuity or visual fields post-operatively. We calculated a specificity of 85% and a negative predictive value of 100% however the authors comment that they did not observe any significant relationship between intra-operative VEP changes and post-operative improvement in visual field defect.

*Kamio *et al. described one case where VEP amplitude decreased. This correlated directly with resecting a piece of tumour adherent to the optic chiasm. Despite halting surgical manipulation and administering methylprednisolone, the VEP waveforms did not improve; the patient experienced complete bi-temporal hemianopsia post-operatively and the resection was sub-total. As this was the only case of VEP amplitude deterioration in the study, both sensitivity and PPV were 100%. For patients who experienced a transient decrease in VEP waveforms, there were no post-operative visual deteriorations; 50% had improved visual outcome and 50% were unchanged. No statistical analysis was performed by the authors.

The study by *Chung *et al. found no association between intra-operative VEP waveforms and post-operative visual acuity or visual fields. Spearman’s correlation analysis was used (P > 0.05). From 95 eyes with reproducible VEP waveforms, 14% demonstrated worsened visual acuity and 14% demonstrated worsened visual fields post-operatively. Whilst this was higher in the group with decreased VEP amplitude (17% of eyes in this group had worsened post-operative visual acuity and 25% demonstrated deterioration in visual fields) it was also noted in the group with improved VEP amplitude (11% in both domains).

The cohort study by *Chacko *et al. reported no cases of worsened post-operative visual outcome in either group (with or without VEP monitoring). They did however report a superior improvement in post-operative visual fields of the test group (with monitoring) compared to the control group, with mean percentage improvement of 12.4% (two sample t-test significant, t = 2.98, p = 0.003). No statistical difference in the improvement of visual acuity between the test group and the control group was found.

*Nishimura *et al. also reported zero incidences of worsened visual outcome. Of the 158 eyes tested, 5% experienced a decrease in VEP amplitude; visual function was reported to be unchanged in all of these (false positives). In the unchanged VEP group, 50% of eyes had improved visual outcome post-operatively; this was 31% in the transient decrease VEP group. Of those with unchanged VEP amplitudes there were no post-operative visual deteriorations (100% negative predictive value).

*Sasaki *et al. reported that 100% of eyes demonstrating a permanent deterioration in VEP amplitude also showed deterioration in post-operative visual outcome whilst 89% of those with stable VEP amplitudes had unchanged visual outcomes. They therefore concluded that the two were well correlated and that in some patients this could avoid or minimize post-operative visual deterioration. Of note, only 1 of the 14 eyes demonstrating a permanent deterioration in VEP was secondary to pituitary adenoma. The only eye which showed an improvement in VEP amplitude and subsequent post-operative improvement in visual function also belonged to a patient with pituitary adenoma and of three eyes with temporary VEP amplitude deteriorations one patient had a pituitary adenoma. For this patient, after decompression of the tumour, the VEP recovered, and the visual function improved.

## Discussion

The first descriptions of VEP recordings date back to 1934 [27]. By the 1960s, VEPs were being utilised as a diagnostic aide in many conditions affecting the optic pathway including multiple sclerosis, compressive tumours, optic atrophy, amblyopia and stroke [28]. Utilisation for intra-orbital surgery was first described in 1973 [2] and although a series of subsequent case-reports and two larger series appeared favourable these were later largely disregarded as anecdotal. *Cedzich *et al. concluded in 1987 that VEP was “too susceptible to non-specific influences” to be a reliable indicator for intraoperative visual change [29]. Interest in the technique rekindled with the observation of improved reliability of VEP recordings under total intravenous anaesthesia (TIVA) as compared to with inhalational anaesthesia [4, 30]. However, its enhanced recordability did not make intraoperative VEP monitoring clinically meaningful yet. Indeed, *Chung *et al. report no association between the intraoperative fluctuation of VEPs and patients’ postoperative visual outcomes [24]. Diverging reports on the usefulness of VEPs have therefore led more recent research to focus on identifying further means of improving their reliability and interpretability: Instead of goggles as a photo-stimulation device, *Sasaki *et al*.* use soft silicone discs that increase the device’s surface application to patients’ eyelids, along with electroretinography (ERG) to ensure that the light stimulus indeed reaches the retina [6]. *Houlden *et al. propose dual intraoperative monitoring of electroencephalography (EEG) and VEPs, based on their observation of improved VEP reproducibility in the presence of low – rather than high – amplitude EEG [9]. *Sato* investigated the impact on VEPs of photo-stimulation parameters – namely the light emission time and amount of light delivered per stimulus – and observed that the cortical wave responses measured following cessation of light stimulation represent a more reliable means of VEP monitoring than the waves measured during photo-stimulation proper [31]. And *Gutzwiller *et al*.* venture that the use of white light flashes, instead of the previously standardly used red light stimuli, may provide better visual field monitoring, as white light not only stimulates cones within the macula, but also the rod-rich regions outside the macula [8].

These advances testify to a renewed, albeit cautious interest in the use of intra-operative VEP monitoring in neurosurgery. Technical considerations remain to be explored; for example, whether a technique-related distinction exists in VEP monitoring for posterior versus anterior visual pathways.

Regarding surgeries involving the anterior optic apparatus, intuitively VEP monitoring is likely best indicated during operations addressing pathology that is adherent to the optic apparatus [10, 11, 13]. Although this can at times also be the case for pituitary adenomas, it is not characteristic. Transsphenoidal procedures are more frequently performed for pituitary adenoma than for any other anterior or central skull base pathology, but it is not clear whether VEP monitoring is of added value during these procedures. Therefore, in this literature review, we have attempted to highlight the information specific to pituitary adenoma alone. Accordingly, our review aimed to determine whether, in patients undergoing transsphenoidal surgery for pituitary adenoma, intraoperative monitoring of visual evoked potentials is a safe, reliable, and effective technological adjunct in predicting postoperative visual function.

### Summary of evidence

We reviewed 11 studies of variable statistical quality (Table 2) and conclude that at present, there is limited low-quality evidence on the safety, reliability, and effectiveness of intraoperative visual evoked potential monitoring in patients undergoing transsphenoidal surgery for pituitary adenoma.

### Safety

There were no cases of operative complications directly related to intra-operative VEP monitoring reported in any of the 11 studies included, therefore suggestive that VEP monitoring is safe in transsphenoidal surgery.

### Reproducibility

All studies used methods such as LED goggles, silicone discs, simultaneous ERG monitoring or TIVA to try and optimise stability and reproducibility and the VEP waveform reproducibility and stability ranged from 83%-100%.

Intra-operative EEG has been shown to “greatly contribute” to intra-operative VEP reproducibility [9] but was only used in one of the studies. Its usage can complement depth of anaesthesia monitoring and facilitate a steady state anaesthesia.

Practical considerations to increase reproducibility not mentioned in the studies include the physical application of the electrodes (placed according to *the ten twenty electrode system of the International Federation* for scalp electrode placement [32]); protection of the visual stimulation to the visual pathways from interference from intraoperative lights (using foil or a black light shield patch) and ensuring a secure fixation of the light stimulating device.

### Effectiveness

The analysis of the technique’s effectiveness is limited by the number of studies available. Furthermore, whilst all but one looked at visual outcome pre- and post-operatively there was variation in the methodology and the outcomes reported. The only comparative study that we found did not look at the association between VEP amplitude and visual outcome but did describe a statistically significant improvement in post-operative visual field testing when VEP monitoring was used. The remainder of the studies varied in their conclusions on the relationship between intra-operative VEP and post-operative visual outcome. Amongst the higher quality studies, *Feng *et al., in their study of 42 patients, described a direct correlation between intra-operative VEP changes and visual fields whereas *Chung *et al. and *Toyama *et al. *–* two studies of similar quality examining 53 and 20 patients respectively – found no significant correlation between VEP waveforms and post-operative visual outcome.

However, given that all studies involved the surgeon temporarily halting surgical manipulation at the point of a VEP amplitude decrease there is an underlying assumption that all teams believed that this may indeed be a contemporaneous warning sign of optic injury. Prolonged latency has also been used as an indicator to the optic apparatus; however, latency is felt to be a parameter that is more difficult to reliably monitor with flash VEP [12] and reported to be difficult to evaluate in the presence of decreased amplitude [6, 11, 12]. Latency was monitored in six of the studies in this review, but no statistically significant conclusions were drawn from these.

There was gross heterogeneity in the statistical analysis performed in each of the studies with only three of the studies allowing sensitivity (i.e., the ability of VEP monitoring to accurately identify intra-operative visual deterioration) to be calculated. Furthermore, due to the small number of true positives across the studies, the sensitivities calculated must be interpreted with caution. Conversely, the high specificity (85–100%) and negative predictive value (90–100%) found in these studies should be recognised and may provide intra-operative re-assurance of good visual outcome in the absence of VEP deterioration.

Accurate contemporaneous interpretation of VEP is variable and the time needed to feed-back to the surgeon will change depending on the specific stimulation rates used and the number of averaged responses recorded. As the technique reaches a greater level of refinement in the future, it is conceivable that it may allow the reliable prediction of increasingly discrete visual deficits that can be considered intraoperatively—in real-time—thereby confirming or altering surgical strategy.

### Limitations

Our review has several limitations. Firstly, study size; with only 11 studies ultimately included, our review is likely underpowered to observe small effect sizes. Secondly, study design; as ten of the studies included were case series with variation in patient selection, methodology and outcomes measurement, a meta-analysis was not performed as it would be unlikely to glean any firm conclusions from such heterogeneous sources. Only 3 studies provided sensitivities for VEP in predicting visual function outcomes; these ranged from 25–100% and as described above, may not be reliable due to the small number of true positives across the dataset. Thirdly, the technical specifications of the monitoring equipment used in the studies varied greatly making generalisation difficult. Finally, as demonstrated in Table 1 despite the inclusion criteria there are very few studies which examine VEP monitoring in patients undergoing transsphenoidal surgery for pituitary adenoma alone; *Chacko *et al. was the only one identified (Table 1). The remainder of the studies also included patients with other pathology such as craniopharyngioma, meningioma, Rathke’s cleft cyst and metastatic disease. In view of this literature review’s focus on VEP monitoring for transsphenoidal pituitary resections only, where possible we have attempted to highlight the information specific to pituitary adenoma alone.

## Conclusions

Whilst there is limited and low-quality evidence surrounding the use of intra-operative VEP monitoring during transsphenoidal resection of pituitary adenoma, our review nonetheless suggests that it is a safe and reproducible technique of seemingly increasing reliability in predicting post-operative visual deficits.

As is the case for other neuromonitoring techniques, its intraoperative interpretation still requires it to be confronted with findings from the surgical field. Whether VEP neuromonitoring helps to achieve more aggressive resections without compromising visual outcome in cases of more expansive tumours has yet to be determined through prospective and comparative case studies. Similarly, future work may identify specific adaptations of the technique allowing to optimise its reproducibility for transsphenoidal surgery, as well as specific alarm thresholds better suited for transsphenoidal procedures in predicting post-operative visual outcome.

## Data Availability

All data generated or analysed during this study are included in this published article.

## Abbreviations

VEP: Visual-evoked potentials
TIVA: Total intravenous anaesthesia
LED: Light-emitting diode
ERG: Electroretinography
EEG: Electroenceohalography
NPV: Negative predictive value
PPV: Positive predictive value
TP: True positive
